# Electromagnetic surgical navigation in patients undergoing mandibular surgery

**DOI:** 10.1038/s41598-021-84129-5

**Published:** 2021-02-25

**Authors:** S. G. Brouwer de Koning, F. Geldof, R. L. P. van Veen, M. J. A. van Alphen, L. H. E. Karssemakers, J. Nijkamp, W. H. Schreuder, T. J. M. Ruers, M. B. Karakullukcu

**Affiliations:** 1 Department of Surgical Oncology, Netherlands Cancer Institute, Antoni van Leeuwenhoek, Plesmanlaan 121, 1066 CX Amsterdam, The Netherlands; 2 Department of Head and Neck Surgery & Oncology, Netherlands Cancer Institute, Antoni Van Leeuwenhoek, Amsterdam, The Netherlands; 3 Department of Head and Neck Surgery & Oncology, Verwelius 3D Lab, Netherlands Cancer Institute, Antoni Van Leeuwenhoek, Amsterdam, The Netherlands; 4 Faculty of Science and Technology, University of Twente, Enschede, The Netherlands

**Keywords:** Oncology, Surgical oncology

## Abstract

The purpose of this study was to evaluate the feasibility of electromagnetic (EM) navigation for guidance on osteotomies in patients undergoing oncologic mandibular surgery. Preoperatively, a 3D rendered model of the mandible was constructed from diagnostic computed tomography (CT) images. Cutting guides and patient specific reconstruction plates were designed and printed for intraoperative use. Intraoperative patient registration was performed using a cone beam CT scan (CBCT). The location of the mandible was tracked with an EM sensor fixated to the mandible. The real-time location of both the mandible and a pointer were displayed on the navigation system. Accuracy measurements were performed by pinpointing four anatomical landmarks and four landmarks on the cutting guide using the pointer on the patient and comparing these locations to the corresponding locations on the CBCT. Differences between actual and virtual locations were expressed as target registration error (TRE). The procedure was performed in eleven patients. TREs were 3.2 ± 1.1 mm and 2.6 ± 1.5 mm using anatomical landmarks and landmarks on the cutting guide, respectively. The navigation procedure added on average half an hour to the duration of the surgery. This is the first study that reports on the accuracy of EM navigation in patients undergoing mandibular surgery.

## Introduction

Computer-aided-design/computer-aided-manufacturing (CAD/CAM) techniques have become routine use in mandibular surgery. In this, a 3D rendered model of the mandible, constructed from a preoperative computed tomography (CT) scan, is used to plan the exact positions of the osteotomies virtually, in advance of the surgery. In this way, adequate tumor resection and an accurate fit of bone segments that will be used for reconstruction after resection can be planned. In order to convert the virtual plan to the patient in the operating room, patient-specific cutting guides and fixation plates are designed. This procedure is costly and time-consuming. In the meantime, there is a change in the tumor size, for which the cutting guide cannot account for during surgery. Therefore, there is a need for a technology that saves preparation time, money and provides flexibility during surgery.

Surgical navigation has been used increasingly and we hypothesize that this technology fulfills the requirements mentioned above. Surgical navigation provides real-time visual feedback about the position of surgical instruments in relation to the patient’s anatomy. This could potentially be used to translate the preoperative planning to the operating room, thereby eliminating the use of cutting guides.

Electromagnetic (EM) navigation is used routinely in neurosurgery or surgery of the sinonasal cavity, but not in mandibular surgery so far: the fact that the mandible is mobile challenges accurate navigational tracking. However, there are studies that evaluated the technology in phantoms and cadavers. In these studies, accuracy measures mostly involve the comparison between the planned and performed osteotomy or the position of the mandibular condyle before and after the osteotomy^1,2^. For example, Peacock et al. report on a < 2 mm difference between preoperatively planned osteotomy and navigated osteotomy and Nova et al. found a difference between navigated condyle position and preoperative condyle position that was also within 2 mm. Bouchard et al. evaluated the target registration error (TRE) of EM navigation on a dissected mandible with a reference sensor secured to a tooth with composite dental material^3^. The tip of the tracked surgical instrument was placed at different locations on the mandible and the difference between the actual and virtual location was measured (TRE was 2.10 ± 0.88 mm). Seeberger et al. evaluated the usability of an EM tracking device in maxillofacial surgery through testing on a phantom skull under operating room conditions. They report on a TRE of 2.1 ± 0.86 mm^4^. With regards to patient studies, there are only a few reports published on studies that use EM navigation in mandibular surgery^5,6^. These studies were focused on condylar repositioning after osteotomies and did not focus on the TRE of the EM navigation. Thus, the accuracy of EM navigation used in patients undergoing mandibular surgery has not been reported so far. Therefore, as a first step of utilizing intraoperative EM navigation, we conducted a study investigating the accuracy of EM navigation in eleven patients undergoing mandibular surgery.

## Methods

**Figure 1:**
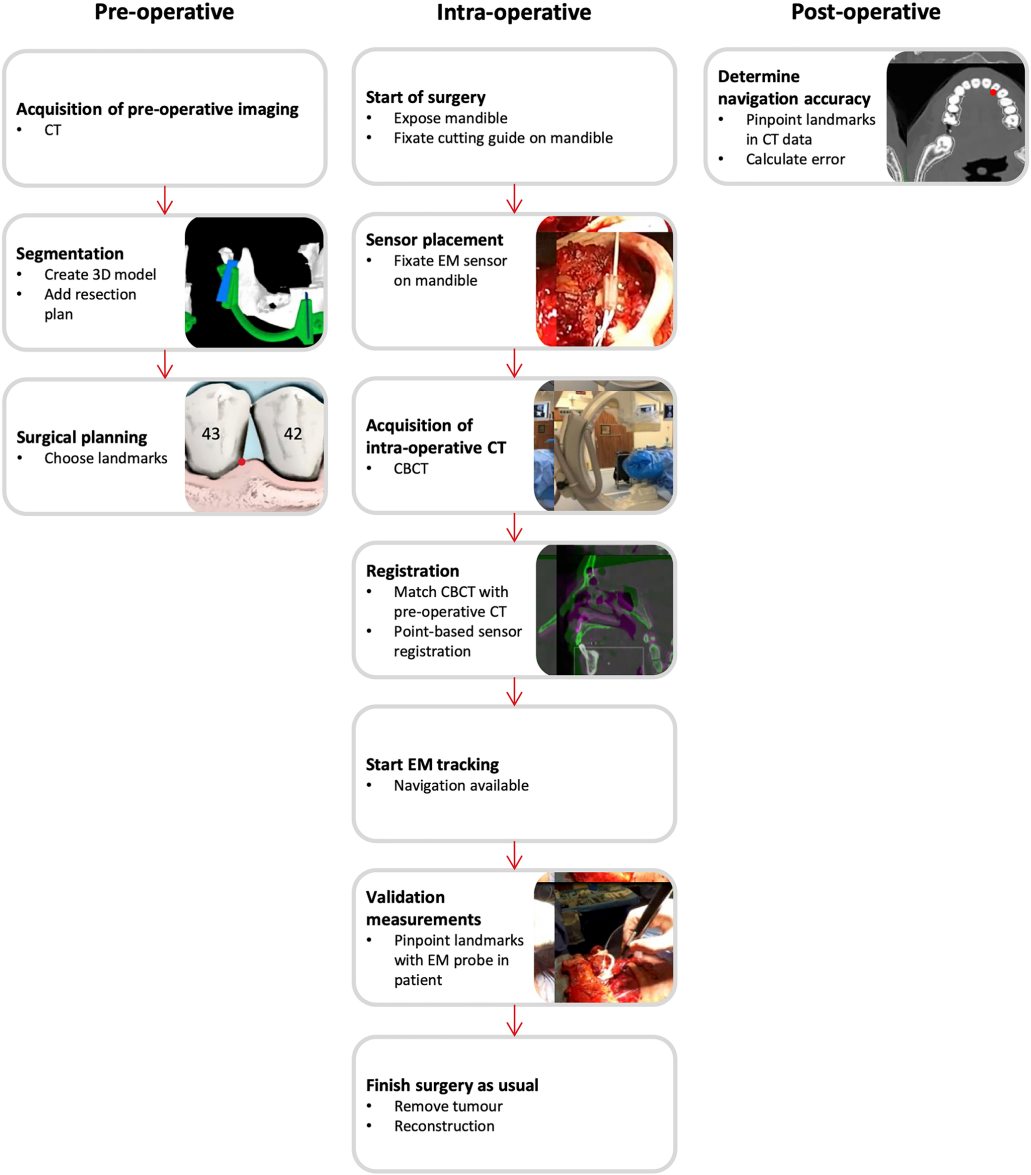
Overview of pre-, intra- and post-operative steps of the surgical navigation procedure.

Patients that were planned for a segmental or hemi-mandibulectomy at the Netherlands Cancer Institute—Antoni van Leeuwenhoek Hospital were included. These patients were treated for malignant and benign tumors invading the mandible, or for osteoradionecrosis of the mandible. The study was approved by the Medical Ethics Committee of the Antoni van Leeuwenhoek hospital and the institutional research ethics board, and all patients signed an informed consent for participation (NL60004.031.17). An overview of the pre-, intra- and post-operative steps of the procedure is shown in Fig. 1.

### EM navigation system

The tracking system (NDI Aurora, Northern Digital Inc., Waterloo, Canada) consists of a field generator, a six degrees of freedom (6DOF) EM-tracked probe and a shielded 6DOF cable sensor. The in-house developed navigation software *SurgNav*^7^*,* holds the registration procedure and a four-display mode (axial, sagittal, frontal and 3D) where the position and orientation of the tracked probe and sensor are visualized relative to the imaging data and the virtual model.

### Pre-operative model

All patients underwent a preoperative conventional diagnostic CT scan (Toshiba Aquilion high resolution helical CT scanner, 1.0 mm slice thickness and 0.468 × 0.468 mm voxel size). Automatic segmentation of the mandible was performed to create a virtual 3D model^8^. The surgeon planned the location and orientation of the resection planes on this virtual model. A patient tailored cutting guide was designed to fit on the mandible and guide the saw to the planned resection planes. After approval of the design of the cutting guide by the surgeon, the cutting guides were printed in 3D (KLS Martin Group, Tutlingen, Germany) in order to accurately translate the virtual plan to the operating room.

### Surgical approach

**Figure 2:**
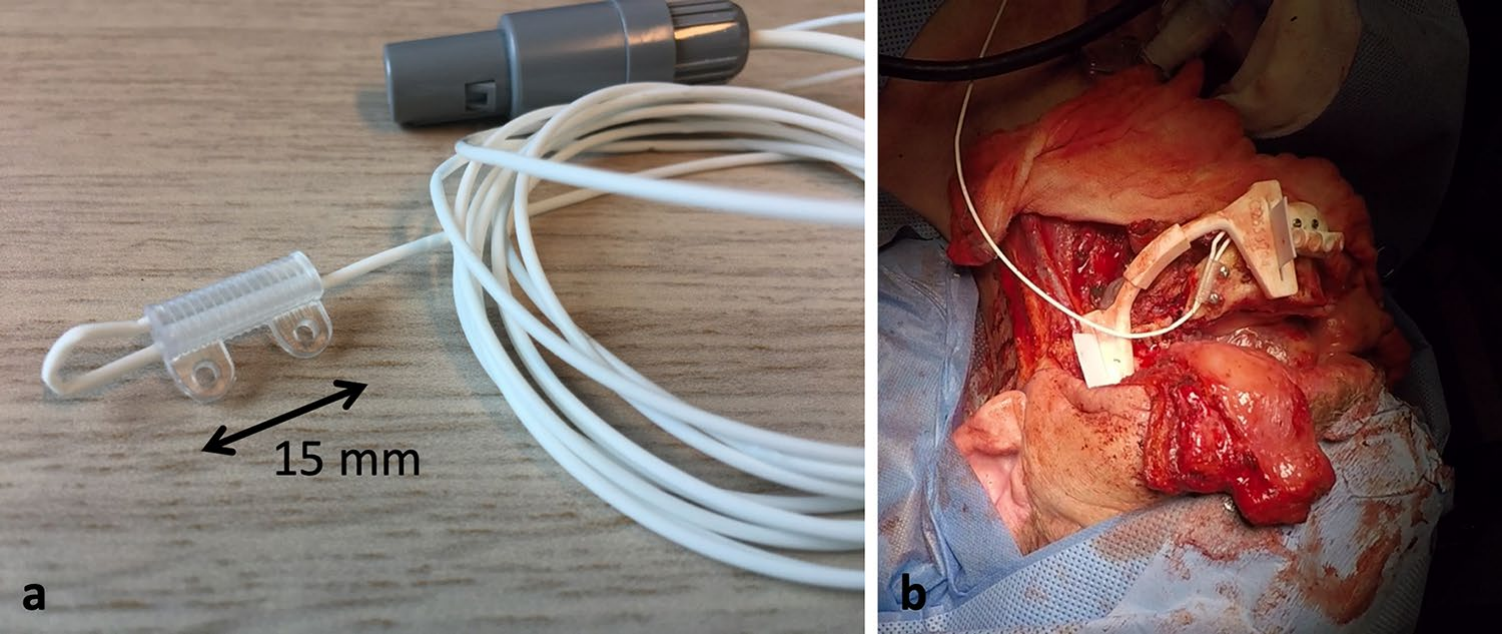
(**a**) EM sensor placed in a sensor housing module that was designed for this study. (**b**) The sensor housing module with EM sensor fixated to the mandible with screws.

During the surgery, the mandibular bone was exposed, and the 3D printed cutting guide was positioned and fixated to the mandible with screws. A dedicated sensor housing module for the 6DOF EM-sensor was fixated to the part of the mandibular bone that was planned for resection (Fig. 2).

### Intra-operative procedure

To register the real-time situation in the operation room with the virtual 3D model, landmarks that are precisely localizable on both the patient and the 3D model are matched. Usually, e.g., in neurosurgery, these landmarks are placed prior to the preoperative CT scan so that they are present in the 3D model. In the current study, the screws of the cutting guide are used as landmarks for registration as a substitute for screws that can be placed before the surgery and intraoperative CBCT is the substitute for preoperative CT with screws in situ. With this approach, we could test if a CT with preoperatively placed mandibular screws can be used accurately for navigation purposes. Therefore, directly after positioning and fixation of the cutting guide and EM-sensor at the mandible, a cone beam CT scan (CBCT) was acquired. This was done in the hybrid operation room, equipped with an Allura FD20 CBCT system (Philips Medical Systems, Best, the Netherlands), with 0.688 mm isotropic voxels. The CBCT scan could be superimposed on the diagnostic CT scan that was used to construct the 3D model, using bone registration in an in-house developed software program. This bone registration was performed using an automatic rigid gray-value registration with mutual information as a cost function, within a user-defined region of interest (ROI) including only the mandible. This way the screws that were visible on the CBCT could be matched with the 3D model to allow registration of the 3D model with the patient in the operation room. Thus, the CBCT scan was only used to register the 3D model with the real-time situation in the operation room, and as a surrogate for preoperative CT with screws planted. An intraoperative CBCT is not compulsory for utilization of the proposed method.

The EM field generator was positioned near the patient’s head. The location of the mandible was tracked by the 6DOF EM tracked sensor that was fixated to the mandible with the sensor housing module. A rigid point-based registration was performed by pinpointing four landmarks (screws of the cutting guide and screws of the sensor housing module) using an EM tracked pointer in the patient and simultaneously indicating the corresponding points in the CBCT scan (that was superimposed on the 3D model). The error of the point-based registration was expressed as fiducial registration error (FRE), which is the root mean squared distance among corresponding landmarks used in registration.

**Figure 3:**
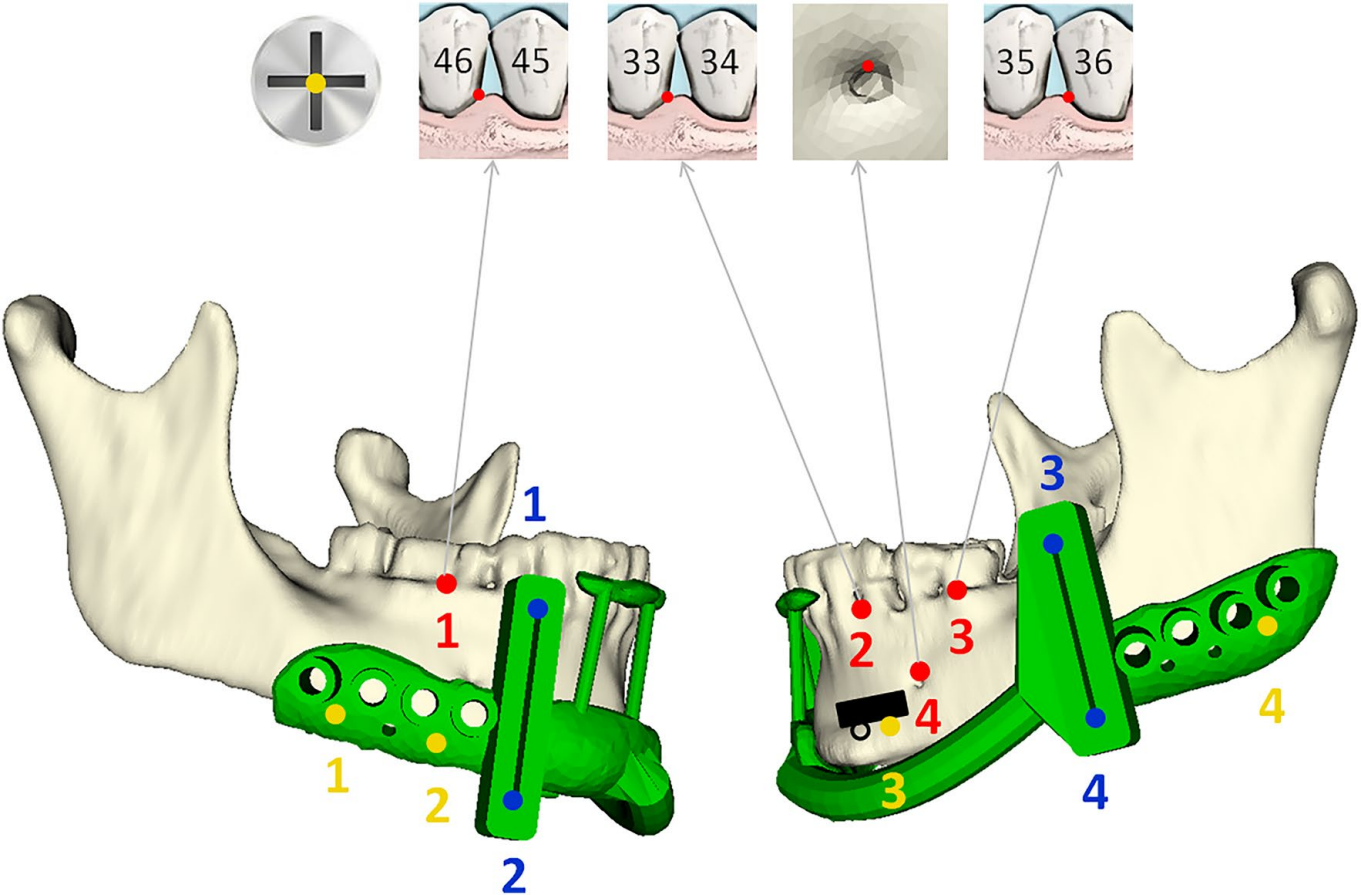
Schematic model of a mandible with the cutting guide (green). The sensor housing module (black) is fixated on the part of the mandible that will be resected. The yellow landmarks are used for fiducial registration. The anatomical landmarks (red) are located on teeth and mental foramen. The landmarks at the cutting guide (blue) are located in the upper and lower ends of the sawing slots. Dental numbering according to the FDI World Dental Federation (ISO 3950).

**Figure 4:**
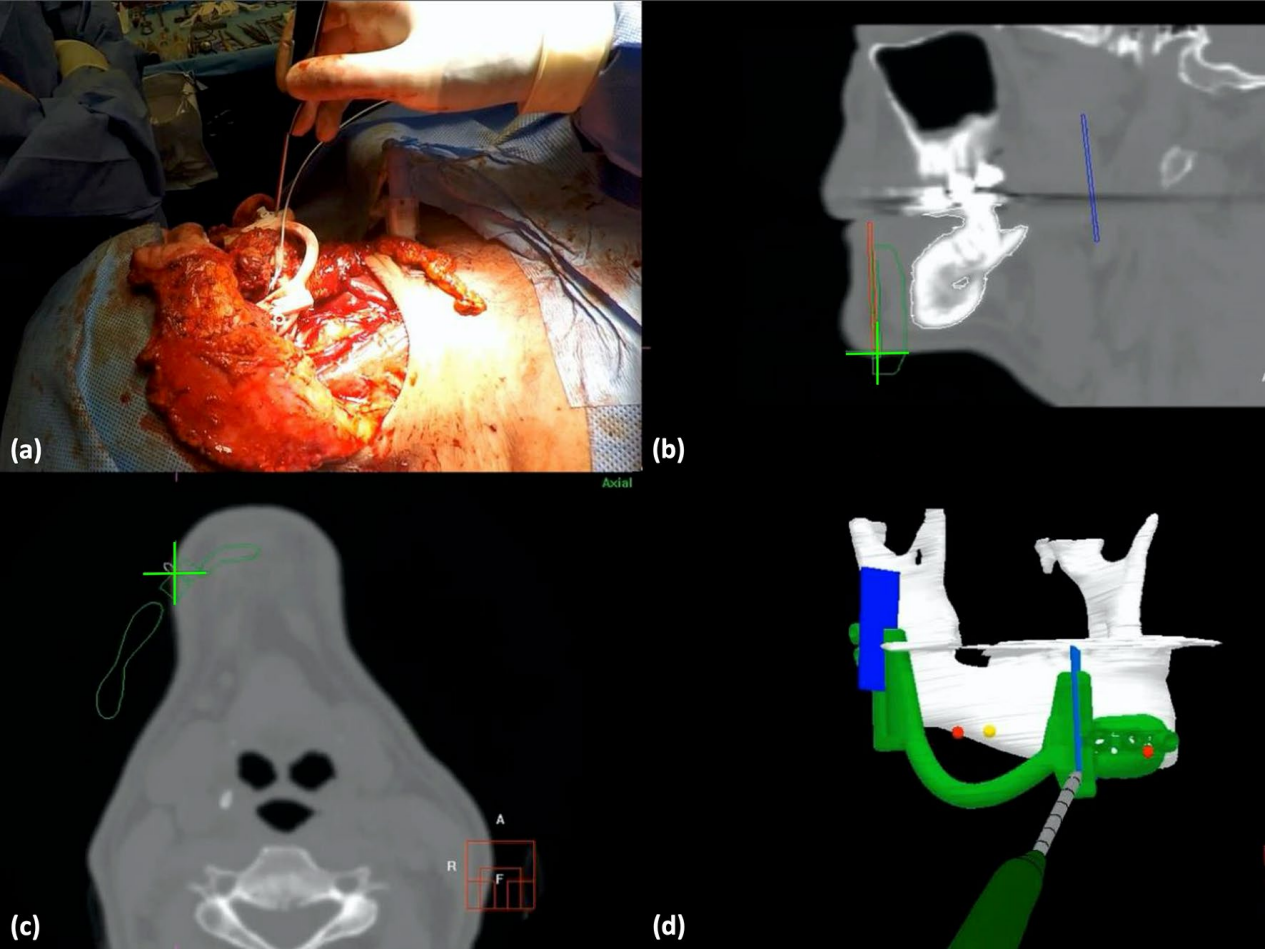
The surgeon points at the resection plane with the tracked pointer (**a**). The position of the pointer in relation to the mandible (white), the cutting guide (green), and the resection planes (blue) are shown in the sagittal and axial sections (**b**,**c**) and the 3D model (**d**).

Accuracy measurements were performed by pinpointing four anatomical landmarks (e.g. mental foramina, cusps mandibular teeth) and four landmarks on the cutting guide (the end points of the sawing slots) (Figs. 3, 4) with the trackable pointer.

### Post-operative evaluation

Two observers indicated the anatomical landmarks, and the landmarks on the cutting guide, in the intraoperative CBCT data. Inter-observer variability was calculated as the intraclass coefficient (ICC). The mean position of the points indicated by the two observers was used as the virtual location of the landmark in the CBCT data. The accuracy of the system was determined by comparing the pinpointed location of the landmarks on the patient with the location of the corresponding landmarks on the CBCT data. The location of the landmarks (in x,y,z-coordinates) that are pinpointed by the trackable pointer on the patient (x_1_,y_1_,z_1_) were saved into the same coordinate system as the locations of the corresponding landmarks on the CBCT (x_2_,y_2_,z_2_). The distance between these two points (one originating from the landmark on the patient (x_1_,y_1_,z_1_) and one originating from the corresponding landmark on the CBCT (x_2_,y_2_,z_2_)) was expressed as the target registration error (TRE), meaning the Euclidean distance between a pair of corresponding landmarks.

### Ethics approval

The study was approved by the institutional ethics committee and was performed in accordance with the ethical standards as laid down in the 1964 Declaration of Helsinki. Please find the approval letter attached, including a translated version.

### Consent to participate

Informed consent was obtained from all individual participants included in the study.

### Consent for publication

Patients signed informed consent regarding publishing their data and photographs.

## Results

### Patients

A total of eleven patients were included in this study. These patients underwent a segmental (*n* = 9) or hemimandibulectomy (*n* = 2) with a free fibula flap reconstruction (*n* = 9), pectoralis major flap reconstruction (*n* = 1) or a deep circumflex iliac artery bone flap reconstruction (*n* = 1), at the NKI-AVL, between December 2017 and September 2019 (Table 1).

**Table 1:** Specifications and pathology outcomes of the surgeries of the patients included in the study.

Case	Gender	Age	Mandibulectomy	Reconstruction	Tumour type/osteoradionecrosis
1	M	74	Segmental	Free fibular flap	T4aN0M0 SCC retromolar trigone right
2	M	48	Segmental	Free fibular flap	T4aN1M0 intraosseous mucoepidermoid carcinoma right
3	M	53	Segmental	Free fibular flap	Osteoradionecrosis
4	M	42	Segmental	Free fibular flap	Osteoradionecrosis
5	M	50	Hemi	Free fibular flap	T4N0M0 SCC retromolar trigone left
6	F	58	Segmental	Free fibular flap	T4aN0M0 alveolar process region 42–37
7	M	65	Segmental	Free fibular flap	T4aN1M0 SCC retromolar trigone right
8	M	64	Segmental	Pectoralis major flap	Osteoradionecrosis
9	M	67	Segmental	Deep circumflex iliac artery bone flap	T4aN2bM0 SCC floor of mouth right
10	M	58	Segmental	Free fibular flap	T4N0M0 SCC midline floor of mouth midline
11	F	76	Hemi	Free fibular flap	T4N0M0 SCC alveolar process right

*F* female, *M* male, *SCC* squamous cell carcinoma.

### Accuracy of the EM navigation system

In seven out of eleven patients, the FRE of the EM navigation was < 1 mm, indicating a very high accuracy of the ready-to-use technology (Table 2). The deviating FRE of patient five was caused by the fact that the sensor was not properly fixated in the sensor housing module.

**Table 2:** The accuracy measures for the registration (fiducial registration errors, FRE) and the navigation procedure (target registration errors, TRE).

Case	FRE (mm)	TRE (mm)	
		Anatomical landmarks	Cutting guide landmarks
1	0.8	2.0	1.4
2	0.5	2.6	1.6
3	1.5	3.3	2.7
4	0.6	2.0	1.2
5	4.1	4.6	3.7
6	0.6	2.2	1.7
7	1.9	4.4	3.1
8	1.6	3.6	1.7
9	0.8	3.2	5.8
10	0.7	4.9	4.8
11	0.7	1.9	1.3
Mean (STD)	1.2 ± 1.1	3.2 ± 1.1	2.6 ± 1.5

*FRE* fiducial registration error, *TRE* target registration error, *STD* standard deviation.

Mean TREs measured on anatomical landmarks and on landmarks on the cutting guide were 3.2 ± 1.1 mm and 2.6 ± 1.5 mm, respectively.

### Inter-observer variability

In order to account for the variability of pinpointing the landmarks on the CBCT, the inter-observer variability was measured. The ICC of the anatomical landmarks, and the landmarks on the cutting guide were for both subsets 1.0, meaning that the landmarks pinpointed on the CBCT by the two different observers resemble each other. This indicates that landmarks were evident on the CBCT.

### Surgical workflow

**Figure 5:**
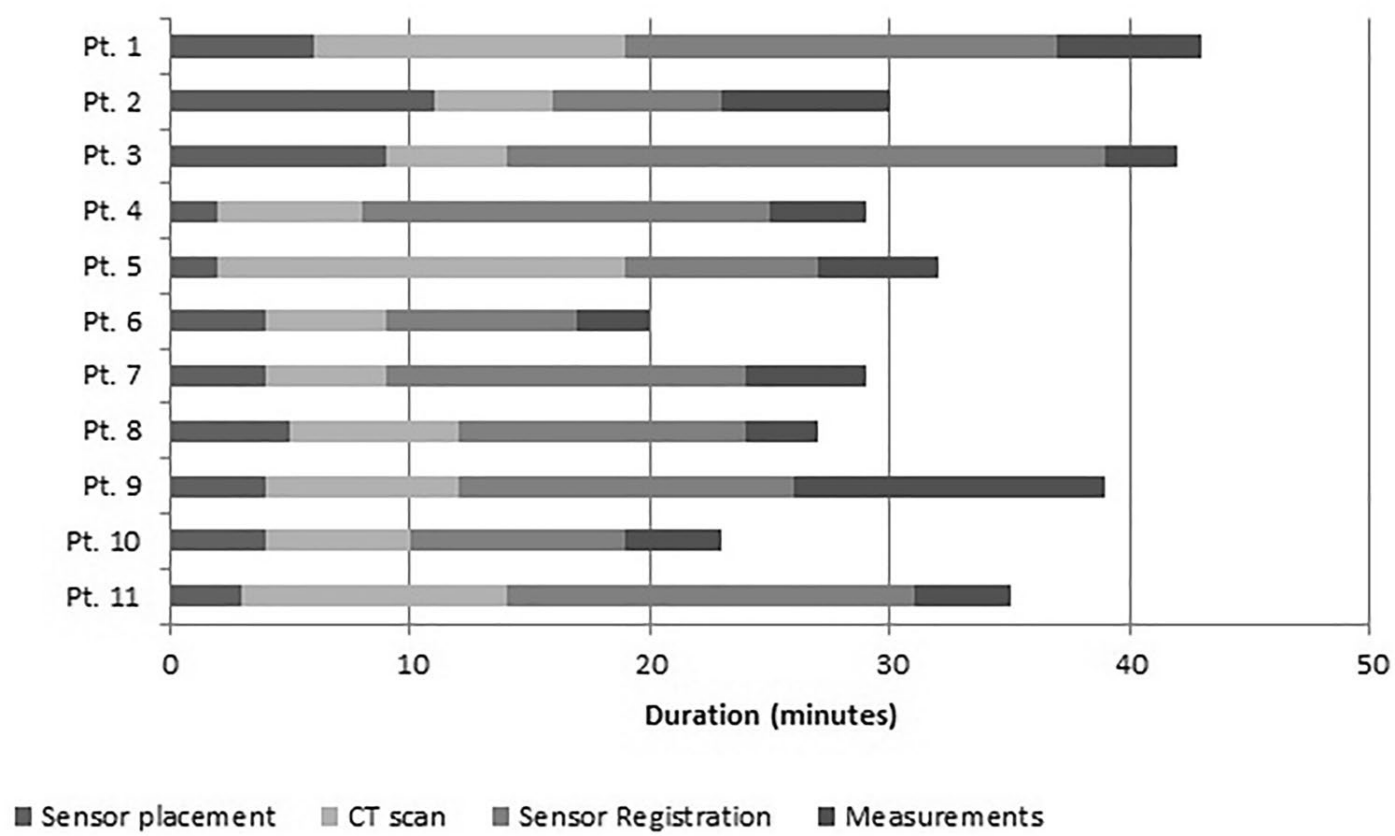
Surgical delay due to navigation workflow.

The total time that was needed to prepare the EM navigation for use, and to perform the accuracy measurements, was on average 32 min (Fig. 5). Registration of the sensor was most time consuming. This step was often redone in order to improve the accuracy of the system.

## Discussion

This study tested EM navigation in eleven patients undergoing mandibular surgery, with the aim to evaluate whether this technology could provide accurate guidance for localization purposes, e.g. tumour margins and planned cutting planes for accurate mandible reconstructions. The accuracy of the EM navigation system was evaluated by measuring the deviation between the location of a landmark that was pinpointed on the patient with a tracked pointer and the location of the corresponding point in the 3D virtual model of the surgical navigation system. The deviation was 2.6 ± 1.5 mm and 3.2 ± 1.1 mm for landmarks located on the cutting guide and for anatomical landmarks, respectively. The navigation procedure added half an hour to the duration of the surgery.

The use of surgical navigation during mandibular surgery could optimize the clinical workflow in several ways. First of all, the surgical navigation can shorten preoperative preparation time. Preparation of the virtual model for surgical navigation takes one day maximally, and only depends on the date of the preoperative CT scan. Thus, if radiology planning allows, preparation can be performed very near to the day of surgery. This procedure eliminates the factor of tumor growth in the time between virtual surgery planning, printing of the patient specific cutting guides, and the surgery. As a result, the virtual model provides a more accurate representation of the real-time situation, which can affect the number of positive bone resection margins. Besides the shorter time needed for preparation, the surgical procedure itself could also be performed faster when reconstruction segments fit the mandibular defect precisely. Accurate guidance for localization of the resection planes could lower the number of cases in which reconstruction segments have to be adjusted manually to fit the mandibular defect, which is a time-consuming process.

In surgical navigation, the real-time position of the patient and the surgeon’s instrument are tracked by the navigation system and registered with a patient-specific anatomical 3D model. Most commercially available surgical navigation technologies use optical tracking technologies to track the patient and the surgical instruments^9^. However, electromagnetic (EM) navigation has practical advances over optical navigation for use during mandibular surgery: patient reference sensors and instrument sensors are small (less than 2 mm in diameter and 1 cm length), and no line-of-sight is required for application in a small surgical field^9^. In addition, the EM field generator can be positioned near the patient’s head, e.g. underneath the operating table.

For mandibular/maxillofacial surgeries specifically, optical or EM navigation has been evaluated in patients or phantoms (Table 3). The different studies use different definitions for the outcome measures. However, all provide some insight in the accuracy that was obtained by using the technology. The table shows that the current study is the first study reporting on accuracies of EM navigation in a group of patients, instead of cadavers/phantoms and that accuracies are comparable to the accuracies obtained with optical navigation that was used in patients undergoing mandibular surgery. This also applies for the accuracies reached in the phantom/model studies. Compared to other applications of the navigation system that was used in this study, the application in mandibular surgery seems quite accurate. For example in malignancies in the pelvis, TREs of 4.0–6.3 mm were reported^7^.

**Table 3:** Overview of studies evaluating surgical navigation for use in mandibular/maxillofacial surgeries.

Author	Year	Navigation	Number of patients	Phantom/model	Outcome	Definition
Hasan et al.^10^	2019	Optical	5	–	1.7 ± 0.8 mm, 5.4 ± 1.5° and 6.7 ± 4.6°, respectively	Difference in distance, pitch, and roll between planned resection plane and performed resection planes
Naujokat et al.^11^	2017	Optical	6	–	1.52 mm	Mean difference between planned osteotomy line and performed osteotomy line
Naujokat et al.^11^	2017	Optical	–	3 skull models	1.03 mm (1), 0.98 mm (2) and 1,7 mm (3)	FRE: based on metal points on the mandibles (1), metal points on a splint based on the occlusal surface of the mandible teeth (2) and anatomical landmarks that were located interdental on the alveolar bone
Shan et al.^12^	2016	Optical	20	–	79% < 1 mm; 87% < 2 mm; 92% < 3 mm	Difference between preoperative plan and postoperative outcome
Lee et al.^6^	2019	EM with real-time augmented model	1	–	1.71 ± 0.63 mm	TRE measured on three condylar landmarks
Berger et al.^5^	2018	EM	10	–	No significant difference	Position of condyles after high oblique sagittal split osteotomy, manually or EM guided; no TRE reported
Nova et al.^2^	2017	EM	–	6 plastic skull models	± 2 mm	Displacement of the condyle between preoperative CT and postoperative CT
Peacock et al.^1^	2015	EM	–	Human cadavers and live minipig	< 2 mm	Differences between the navigation’s prediction of the location of the osteotomy and the virtual planning
Bouchard et al.^3^	2012	EM	–	3 minipig cadavers	2.35 ± 1.35 mm	Mean difference in width mandibular rami after osteotomy and in the 3D model
Bouchard et al.^3^	2012	EM	–	1 dissected mandible	2.10 ± 0.88 mm	TRE: The pencil tip was placed in different holes on the mandible and the difference between the actual and virtual location was measured in millimeters (n = 11 measurements)
Seeberger et al.^4^	2012	EM	–	Plastic skull phantom (maxilla)	2.1 mm ± 0.68	TRE
The current study	2019	EM	11		1.2 ± 1.1 mm 2.6 ± 1.5 mm 3.2 ± 1.1 mm	FRE TRE measured on cutting guide landmarks TRE measured on anatomical guide landmarks

*EM* electromagnetic; *TRE* target registration error; *FRE* fiducial registration error.

Sun et al. argued that inaccuracies of 1.5 mm can be considered as clinically acceptable^13^. Thus, with inaccuracies of 2.6 and 3.2 mm (TRE) as reached in the current study, there is still room for improvement. The TRE is a result of a couple of factors: the FRE, the variability in pinpointing the validation landmarks on the patient and selecting the corresponding landmarks on the CBCT and, an inadequate fixation of the sensor onto the mandible. Within the following paragraphs, these different factors will be discussed on how the inaccuracy was minimized in the current study and suggestions will be done on how to minimize the inaccuracies further for future research.

The accuracy of the registration between the patient during surgery with the virtual model, is expressed as the FRE (1.2 ± 1.1 mm). This value depends on several factors: the accuracy of the NDI Aurora EM tracking system, the accuracy of the CBCT-CT registration and the variability in pinpointing the landmarks used for fiducial registration (both on the patient and on the CBCT). According to the manufacturer, the used NDI system had a tracking accuracy of 0.8 mm in a laboratory setting. This inaccuracy can increase in the presence of any metal distortions in the EM field. However, Seeberger et al. showed that electromagnetic interference due to metallic instruments was significant, but that the effect on the TRE was still acceptable in comparison to optical navigation devices^4^. Inaccuracies of the CBCT-CT registration could be a result of a change in bone anatomy in the time between the pre- and intra-operative CT. However, since this registration is automated on bone contours and the result is checked visually, no large inaccuracies are expected in this step of the procedure. For fiducial registration, the head of the screws used for the cutting guide were used. These were clearly identifiable on the patient and also on the CBCT. The slice thickness of the CBCT was 0.7 mm, which could have contributed to the inaccuracy of this particular step in the procedure.

With regards to the landmarks used for fiducial registration, instead of using screws on the cutting guide as was done in this study, Lee et al. used an, especially for this purpose designed, registration body. This registration body was made as a symmetrical arch that could be fixated to the mandible. It had 24 holes of different depths and in different positions, containing 1 mm in diameter ceramic balls for use as fiducial points for registration. Using this registration body, they obtained a registration accuracy of less than one mm^6^. This is most likely caused by the fact that a registration body allows an optimal configuration, i.e. a larger distance between the locations of the fiducial registration landmarks. It is shown that a larger distance between fiducial registration landmarks increases the registration accuracy^14^. Also in optical navigation, the use of a splint resulted in an increase in accuracy: Naujokat et al*.* evaluated different registration methods for the mandible by using three skull models and infrared navigation^11^. Registration was performed with metal points on the mandibles^1^, metal points on a splint based on the occlusal surface of the mandible teeth^2^ and anatomical landmarks that were located interdental on the alveolar bone. The FREs found were 1.03 mm, 0.98 mm and 1.7 mm, respectively. Although, the results of these studies were obtained in one patient and in models, respectively, we can still learn from these studies. In seven out of the eleven patients, our FREs were also smaller than 1 mm, and thus, a very accurate registration of the 3D model with the situation in the OR was obtained. However, the FRE could potentially reduce further by using a registration body that increases the distance between fiducial registration landmarks.

The FRE should be as small as possible in order to obtain a small TRE. Therefore, in case the point-based registration resulted in a large FRE, it was advised to redo the registration procedure. The difference between the TRE measured with the anatomical landmarks and the landmarks on the cutting guide can be explained by the variability in pinpointing the anatomical landmarks. Apart from the mental foramen and teeth, there are no precise landmarks identifiable on the mandible. Still, also these anatomical landmarks are multi-interpretable when aiming for a < 1 mm accuracy. Variability in pinpointing the landmarks on the CBCT was minimal, since the ICC for the two observers in selecting both the anatomical and cutting guide landmarks on the CBCT were 1.0. The TRE can also be a result of inadequate fixation of the sensor to the mandible. Rotational movements of the sensor can occur within the sensor housing module, when the mandible was repositioned during pinpointing the landmarks. We are currently working on a new housing module that provides a better fixation and that is smaller (max 10 mm in length). As this was a study reporting on the first results obtained in using surgical EM navigation in a small group of patients, this will be taken into account for improvements in future research.

As the first results on accuracy were promising, we are currently working on methods to optimize the workflow, e.g., to eliminate the need for an intraoperative CBCT for registration, or preoperative CT with screws in situ. The first results for an alternative method for registration are recently published^15^ and with the final aim to use a navigated cutting guide for optimal positioning and guidance during sawing we recently published a paper on a prototype of a navigated cutting guide^16^.

This study evaluated the accuracy of an in-house developed EM navigation system, as a first step towards EM navigated assisted bone resection in mandibular tumour surgery. In a total of eleven patients, EM navigation approaches clinically acceptable accuracies to guide resections in mandibular surgery. Future studies will aim to improve accuracy and practical workflow further. Our group is currently working on an improvement of the registration method and on the implementation of the angle of the resection plane, besides the position of the osteotomy only.

## Data Availability

The dataset generated during and analyzed during the current study are available from the corresponding author on reasonable request.
